# Comparison of ureteroscopy (URS) complementary treatment after extracorporeal shock wave lithotripsy failure with primary URS lithotripsy with holmium laser treatment for proximal ureteral stones larger than10mm

**DOI:** 10.1186/s12894-021-00892-7

**Published:** 2021-09-13

**Authors:** Feng Yao, XiaoLiang Jiang, Bin Xie, Ning Liu

**Affiliations:** Department of Urology, People’s Hospital of Chongqing Banan District, Chongqing, 401320 China

**Keywords:** Ureteral calculus, Ureteroscopy, Lithotripsy, Lasers, Solid-State, Therapeutics

## Abstract

**Background:**

To compare ureteroscopy (URS) complementary treatment following extracorporeal shock wave lithotripsy (SWL) failure with primary URS lithotripsy for proximal ureteral stones > 10 mm, and try to find out acceptable number of SWL sessions followed by safe URS.

**Methods:**

This was a retrospective study following approval from Medical Ethics Committee of People's Hospital of Chongqing Banan District. Patients (n = 340) who received URS in our hospital for stones > 10 mm from Jan 2015 to June 2020 were divided into two groups according to their previous SWL history. Group 1 consisted of 160 patients that underwent unsuccessful SWL before URS. Group 2 encompassed 180 patients without SWL before URS. Patient’s operative outcomes were compared. A logistic regression and receiver operator characteristics (ROC) were used to identify the acceptable number of SWL sessions prior to URS, regarding the intra-operative complications of URS.

**Results:**

The group 1 required more surgery time (41.38 ± 11.39 min vs. 36.43 ± 13.36 min, p = 0.01). At the same time, more intra-operative (68.1% VS 22.8%, p < 0.05) and post-operative (35% VS 18.0%, p = 0.001) complications occurred in group 1. Need more hospital stay in group 1 (2.7 ± 1.2 days vs 1.6 ± 1.1 days, p < 0.05). More patients in group 1 need further URS (16.3% VS 8.9%, p = 0.029). After second URS, the SFR of URS in two groups was insignificant differences (82.5% VS 88.9%, p > 0.05). The median (25–75%) of SWL sessions before URS was 2 (1–3) in group 1. According to the results of logistic regression analysis, patients suffered more SWL failure have an increased risk of complications during URS (OR = 1.995, 95% CI: 1.636–2.434). ROC showed that the optimal number of SWL session followed by URS were 0.5, with a sensitivity of 67.7% and specificity of 71.5%. Intra-operative complication rates of URS treatment were higher in patients who suffered > 1 SWL failure (72.6% vs 57.4%, p = 0.047).

**Conclusion:**

There was no acceptable number of SWL sessions that could be followed by URS with fewer intra-operative complications. Patients who underwent previous SWL were likely to suffer more intra-operative complications, the average operating time, hospitalization time, and needing further treatment, during URS treatment for proximal ureteral stones larger than 10 mm.

## Background

Ureteral calculus is a highly prevalent disease worldwide with a high recurrence rate [1, 2]. Urolithiasis shows variability according to geography, ethnicity, climate, diet and genetics [3]. The occurrence of ureteral stones typically requires ureteroscopy (URS) or extracorporeal shock wave lithotripsy (SWL). SWL has represented the major therapy for ureteral calculi since the 1980s [4]. In comparison to URS, SWL is minimally invasive, has shorter treatment times, fewer complication rates and is cheaper. However, high rates of retreatment are often required, representing a significant weakness in SWL. With advancement in endourology and minimally invasive surgeries with high success rates, the applicability of SWL has decreased. Since 2015, treatment recommendations of the European Association of Urology (EAU) regarding proximal ureteral stones larger than 10 mm have shifted to rigid URS endourologic procedures. SWL has therefore lost its position as a first-line modality, despite its effectiveness [5]. The availability of the equipment dictates the treatment choice, which is often selected based on individually and patient preference. So, in many developing countries, SWL still remains the first-line choice due to its low costs. In our research center, each SWL session costs about 800 RMB, and URS need about 7500 RMB.

It was reported that the stone-free rate (SFR) of re-treating ureteral calculi with SWL decreases significantly after the initial treatment [6]. When SWL fails, URS becomes the most commonly used treatments. But some studies mentioned that more complications during URS procedures were seen in patients having already undergone SWL [7, 8]. All these reports reminded us whether there had a number of SWL sessions followed by safe URS especially for large calculus. We performed the study aiming to compare the URS outcomes between complementary URS treatment following SWL failure with primary URS lithotripsy for proximal ureteral stones > 10 mm, and try to find out the acceptable number of SWL sessions followed by safe URS.

## Methods

### Study population

This was a retrospective study performed from January 2015 to June 2020 following approval from Medical Ethics Committee of People's Hospital of Chongqing Banan District. A total of 340 patients with a single side proximal ureteral stones > 10 mm received URS treatment in our hospital. Group 1 (n = 160) consisted of patients with previous SWL for stones. Group 2 (n = 180) consisted of patients without SWL prior to URS. In our institute, the diameter of proximal ureteral stones less than 15 mm, SWL will be an optional treatment. The treatment procedure was chosen on the basis of the patients’ preference.

Kidney function was assessed prior to all surgeries. Patients also received urinalysis kidney-ureter-bladder (KUB) radiography, computerized tomography (CT) and ultrasonography assessments. The proximal ureter was defined as the area of the ureter between the ureteropelvic junction and the upper border of the sacroiliac joint. Exclusion criteria included patients with renal dysfunction, those with an abnormal anatomy, kidney stones, kidney infection, previous URS, Percutaneous nephrostomy, laparoscopy, or open surgical ureterolithotomy. Blood cell counts, biochemical analysis, urinalysis, and aerobic urine cultures were performed post-operation. When present, urinary infections were treated and tested to confirm sterility. All patients were administered antibiotics at induction during operation. Patients with surgical contraindications were not observed. Auxiliary equipment, such as stone baskets and ureteral balloon dilator, were used in the study.

Complications that occurred intraoperatively were graded according to the modified Satava classification system [9]. Those that occurred after operation were classed according to the modified Clavien classification system [10].

### SWL procedures

We performed SWL as an outpatient procedure using the Dornier Compact Sigma (EMSE 140f, Dornier MedTech, Germany). Shock waves were delivered at a rate of 60 impulses per minute. The maximum number of shock waves per session was 2000–2500. Pulse levels were set to 4–6. KUB radiography and ultrasound were performed to evaluate stone-free status and hydronephrosis after each session at 1–2 week. SWL failure was considered when the stones persisted or when an increasing degree of hydronephrosis was detected. Prior to the SWL procedure, Ureteral J stent insertion was not performed in any case.

### Ureteroscopy technique

URS procedures were performed under general anesthesia using a 8/9.8F and/or 6/7.5F rigid URS (Richard Wolf, Knittlingen, Germany) with a holmium laser (Generally, 0.5 to 1 J of energy per pulse with a pulse frequency of 10 to 15 Hz). Usually, 10 W [1 J × 10 Hz] and 7.5 W [0.5 J × 15 Hz] settings of holmium laser were performed to break stone dusting and getting popcorn effects. Guidewires (0.035 inch) were placed into the ureteral orifice through the ureteroscope which was delivered directly to the stone following guidewires guidance. The ureteral orifice and ureteral strictures were balloon dilated and stone baskets were employed to subvert stone migration into the renal pelvis. Larger fragments were removed through stone retrieval. If a false intramural route formation, bleeding making the surgical field invisible, inability to reach stone or residual fragments larger than 6 mm, double J stents were implanted, and secondary URS was performed after 2–4 weeks. Percutaneous nephrostomy was performed upon urinary tract perforation occurred, and secondary URS was needed to remove ureteral stones.

Considering a large volume of stones located in the proximal ureter, ureteral injury and edema after operation, double J stents were implanted into all patients in our study. Stents were removed 2 to 4 weeks after surgery. Prior to J stent removal, residual stones were examined by KUB. When the residual stones in the ureteral were larger than 6 mm, second URS sessions were performed. Flexible ureteroscopy procedures were performed when larger than 6 mm residual stones migrated to the renal pelvis. All surgeries were performed by urology surgeons with more than 5 years of ureteroscopy experience. The patient could only be discharged without renal colic, fever and gross hematuria. All 340 patients received oral α-receptor blockers to assist the excretion of stones, and the absence of stones was followed up for 3 months. The SFR (3 months after the last treatment) was determined on the basis of KUB, and any calculi residual fragments were considered as a failure for stone free.

### Statistical analysis

Data were analyzed using SPSS25.0 software. Kolmogorov–Smirnov and Probability plots were constructed to verify the normality of the continuous variables. Data are shown as the mean ± SD. The number of cases and percentages were recorded for normally distributed and categorical variables. Intergroup analysis of the continuous variables was performed using a Student-t test. Categorical variables were analyzed with a Chi square test. A logistic regression and receiver operator characteristics (ROC) were performed to identify the acceptable number of SWL sessions followed by safe URS, regarding the intraoperative complications of URS. p values < 0.05 were considered significant.

## Results

We included 340 patients in the study. Patients were sub-divided according to SWL history (group 1: 160 patients and group 2: 180 patients). Group 1 encompassed patients with previously unsuccessful SWL prior to URS. Group 2 consisted of patients without SWL prior to URS for the stone. There were insignificant differences between groups in terms of clinical and stone characteristics (p > 0.05) (Table 1).

**Table 1:** Comparison in terms of clinical and stone characteristics

	Group 1	Group 2	p value
No. patients, n	160	180	
Age, years, mean (SD)	45.2 (8.8)	46.8 (7.6)	0.083
*Gender, n (%)*			
Male	76 (47.5)	88 (48.9)	0.442
Female	84 (52.5)	92 (51.1)	
BMI, mean (SD)	24.39 (3.02)	24.68 (2.68)	0.39
*Side, n (%)*			
Right	68 (42.5)	88 (48.9)	0.142
Left	92 (57.5)	92 (51.1)	
Stone size, mm, mean (SD)	12.6 (1.42)	12.4 (1.39)	0.898
Calcium oxalate stones, n (%)	136 (85)	157 (87.2)	0.331
CT value, Hu, mean (SD)	718.65 (148.30)	739.11 (109.78)	0.159

*URS* ureteroscopy, *BMI* body mass index, *CT* computed tomography, *SFR* stone-free rate

During URS procedure, endoscopic observation revealed edematous inflammation around ureteral calculus, which was defined as impacted ureteral stones. Compared with group 2, group 1 had more impacted ureteral stones (p < 0.05). Most of the impacted ureteral stones were at the original site, only 2 was ureteral steinstrasse. Most of findings in the two groups were significant differences (Table 2).

**Table 2:** Comparison in terms of URS treatment

	Group 1	Group 2	p value
Impacted ureteral stones fund during URS, n (%)	79 (49.4)	30 (16.7)	< 0.05
Energy used for gravel, KJ, mean (SD)	1.459 (0.546)	1.545 (0.606)	0.203
Operation time, minutes, mean (SD)	41.38 (11.39)	36.43 (13.36)	0.01
Using stone baskets, n (%)	113 (70.6)	97 (53.9)	0.001
*SFR of URS, n (%)*			
After first URS session	107 (66.9)	148 (82.2)	0.001
After second URS session	132 (82.5)	160 (88.9)	0.084
Hospitalization times, days, mean (SD)	2.7 (1.2)	1.69 (1.1)	0.02

*URS* ureteroscopy, *SFR* stone-free rate

The SFR in group 1 (66.9%) were significantly lower than in group 2 (82.2%) after the first URS session. Deal with some Satava II complications, more patients in group 1 need second URS (26 VS 16; 16.3% VS 8.9%, p = 0.029). And15.6% patients in group 1 VS 6.7% patients in group 2 (p = 0.007) successfully carried out further URS treatment after placed a double J stent for 2 to 4 weeks. Following the second URS session, the SFR of two groups showed insignificant differences (82.5% VS 88.9%, p > 0.05).

Significant differences in intraoperative and postoperative complications were observed between groups (p < 0.05). In Satava I, bleeding and proximal stone migration occurred (p < 0.05). In Satava II, bleeding making the surgical field invisible and large levels of stone migration to the renal pelvis were observed (p < 0.05). There were no significant differences in Satava III (Table 3).

**Table 3:** Intra-operative complications of URS

Complications	Group 1 (n = 160), n (%)	Group 2 (n = 180), n (%)	p value
**Intra-operative complications**	109 (68.1)	52 (28.9)	< 0.05
*Satava I (Observation)*	56 (35.0)	20 (11.1)	< 0.05
Bleeding nearly no effect to URS operation	29 (18.1)	10 (5.6)	< 0.05
Mucosal tears	6 (3.8)	2 (1.1)	0.107
Stones less than 6 mm migration Renal pelvis	21 (13.1)	8 (4.4)	0.004
*Satava II (requiring endoscopic retreatment)*	52 (32.5)	32 (17.8)	0.001
Mucosal injury (A false intramural route formation)	3 (1.9)	1 (0.6)	0.269
Bleeding making the surgical field invisible	12 (7.5)	4 (2.2)	0.020
Inability to reach stone requiring secondary URS	1 (0.6)	2 (1.1)	0.544
Residual fragments larger than 6 mm	10 (6.3)	9 (5.0)	0.395
Stones larger than 6 mm migration renal pelvis requiring flexible URS	24 (15.0)	15 (8.3)	0.040
Ureteral perforation requiring percutaneous nephrostomy	2 (1.3)	1 (0.6)	0.456
*Satava III (requiring open surgery)*	1(0.6)	0	0.471
Urethral stricture	0 (0)	0	
Ureteral avulsion	0 (0)	0	

*URS* ureteroscopy, *UTI* urinary tract infection

Post-operative complications in group 1 was significantly higher than group 2 (35.0% VS 18.0%, p = 0.001). Fever and hematuria in Clavien I were the most frequent postoperative complication in group 1 (9.2% and 20.0%) and group 2 (3.5% and 15.5%) (p < 0.05). There were no significant differences in Clavien II to V.

**Fig. 1:**
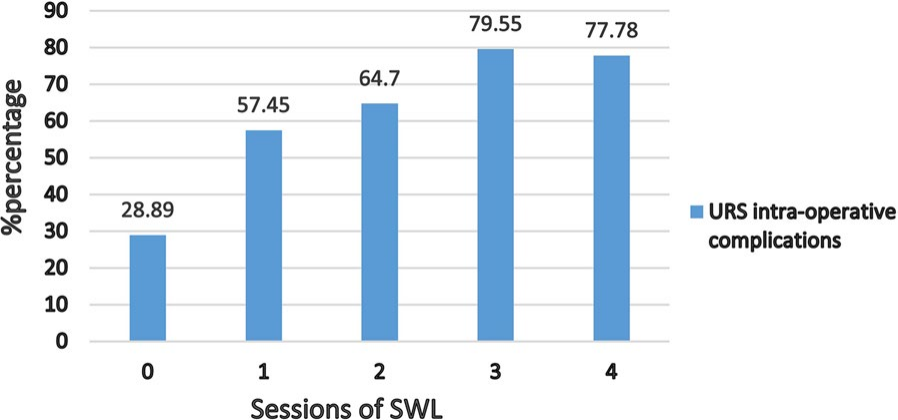
The percentage of URS intra-operative complications in patients with previous different SWL sessions

All 340 participants, which underwent 0, 1, 2, 3, and 4 SWL sessions were 180, 47, 51, 44, and 18, respectively. And, the number of URS intraoperative complications in patients who suffered 0,1, 2, 3, and 4 SWL sessions were 41, 27, 33, 35, and 14, respectively (Fig. 1).

**Fig. 2:**
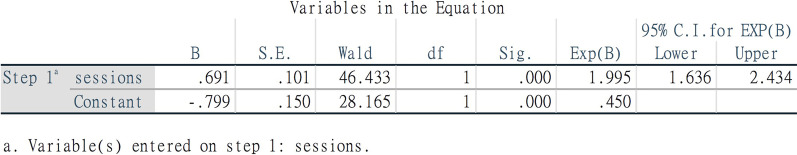
Logistic regression analysis of the number of SWL sessions to URS intra-operative complications

According to the results of logistic regression analysis, patients suffered more SWL failure have an increased risk of complications during URS (OR = 1.995, 95% CI: 1.636–2.434) (Fig. 2).

The median (25–75%) of SWL sessions was 2 (1–3). ROC showed that the optimal number of SWL sessions followed by safe URS were 0.5, with a sensitivity of 67.7% and specificity of 71.5%. Intraoperative complication rates of URS treatment were higher in patients who suffered > 1 SWL failure (72.6% vs 57.4%, p = 0.047).

## Discussion

SWL is both non-invasive and more practical than URS, and is widely used in clinical treatment of urinary calculi. In studies by Ziaee et al., patients with proximal ureteral stones that were 10 to 15 mm in size underwent three SWL sessions and achieved 78.6% SFR, which were higher than the 72.5% SFR of URS (p > 0.05) [11]. Many shortcomings do however remain for SWL, including the requirement for multiple treatment protocols. The success of SWL has been linked to a number of factors including stone size, location and Hounsfield units, as well as technique and distance of skin to stone [6]. Kenneth et al. showed that the SFR of initial SWL treatment was 68%, which decreased to 46% after re-treatment 1, and they observed a further decrease in the stone-free rate after re-treatment 2 to 31% (p = 0.001) [6]. SWL and URS are the most commonly used treatment modalities for proximal ureteral stones [12]. And in cases of SWL failure for proximal ureteral stones, complementary URS treatment is a common procedure.

SWL treatment when compared to URS is considered a less invasive treatment option for ureteral stones. However, SWL can cause tissue damage in the kidney, proximal ureter and adjacent organs [13–15]. The mucosal layer of the ureter was damaged by SWL through the increased number of transitional cells during cytological examination of the urine [13]. The inflammatory response of the target tissues was triggered by shock waves through the release of oxidants, prostaglandins, COX-2 and TNF-α leading to tissue damage and edema [14, 15]. Information on edema in the urothelial mucosa can lead to the small vessels mucosa becoming more fragile, leading to lower levels of hemorrhage. Edema in the urothelial mucosa also complicates subsequent ureteral URS surgery. Holland et al. and Grasso et al. also reported that stone fragments can become embedded in the ureteral mucosa after SWL, occasionally becoming completely submucosal and eventually leading to ureteral stricture [16–18]. All these changes may cause more impacted stones. Severs studies have shown the phenomenon that the impacted stone rates are significantly higher after unsuccessful SWL [19–21], which may cause difficulty and even more complications during URS. In our study, we also found that impacted stones and ureteral inflammation edema were seen predominantly in patients having already undergone SWL.

Complications of URS can occur due to many variables. The size of the stones, stone location, and the size of the ureteroscope used are closely related to the occurrence of complications [8, 22]. In studies by Kilinc et al. [20] and Tugcu et al. [23], insignificant differences were observed between the complication rates of the two groups. However, only proximal and distal-ureteral small stones were investigated in their studies. In Bora et al. study [24], the mean proximal ureteral stone burden was 83.7 ± 57.2 mm^2^ and complication rates were significantly higher in patients with previous SWL. The results of this study similarly showed that the complication rates of URS treatment were significantly higher in patients who suffered SWL treatment failure for proximal stones > 10 mm.

In this study, it been found that impacted stone fragments and friable ureter mucosa lead to bleeding easily during URS treatment for patients suffering SWL failure. For improved visibility of the stones, we applied high-pressure saline injections which led to stone/fragment migration to the renal pelvis. To reduce migration, stone baskets were used in both groups. Although a higher number of stone baskets were used in group 1, patient numbers were significantly higher (15.0%) and required flexible ureteroscopes for further treatment to achieve a stone-free status after 2 weeks. Perforation of the ureter is a serious complication during URS procedures [8, 25]. Alapont et al. showed that 68% of their ureteral perforations occurred with stones > 10 mm [25]. Recent research found that the proximal ureter had lower levels of tensile strength which caused ureteral perforation more likely to occur [26]. When stones baskets or balloon dilatation were performed, avulsion and ureteral perforation can occur, particularly in the upper third of the ureter possessing the impacted stones [27, 28]. In this study, perforation was more common in those undergoing SWL failure, despite the differences lacking significance. According to our experience, maintaining good visibility represented an effective method to reduce perforation.

Kilinc et al. [20] and Tugcu et al. [23] respectively reported that URS procedures after SWL failure require longer operation times than direct URS for both distal and proximal ureteral stones. Our results were in accordance with these data. Due to more impacted stones and significant ureteral inflammatory edema, when fragmenting stones occurred, the time taken for laser assessments was extended and the need for extra manipulations and the use of auxiliary equipment prolonged the URS procedure.

Fever and hematuria were the most frequent postoperative complications, which were significantly higher in the previously unsuccessful SWL group. We consider that these complications may be caused by severe ureteral inflammation and injuries. We did observe only cases of sepsis in group 1. The occurrence of fevers and sepsis resolved with antibiotic and antipyretic drugs. Further complications in the unsuccessful SWL group may lead to longer hospitalization times.

In previous studies, the success rates of URS with or without unsuccessful SWL were not statistically significant. The success rates of URS sessions after SWL failure ranged from 73.6%-78.9% [19–21]. In their researches, the stones were less than 10 mm in diameter. It was also reported that the success rates (72.5%) of URS were lower for stones rang from 10 to 15 mm [11]. In our studies, after one URS session, the SFR in group 2 was 82.2% which was in accordance with the 82% SFR of primary URS in the proximal ureteral for stones larger than 10 mm [29]. However, group 1 (66.9%) was significantly lower than group 2. To deal with Satava II intra-operative complications, a double J stent was placed and part of these patients required secondary URS treatment. Following the further URS procedure, the SFR of the two groups showed no significant differences (82.5% VS 88.9%). Although there was insignificant difference in SFR, patients who suffered SWL failure were more likely to need multiple URS treatment.

In developing countries such as China, a large number of treatment centers lack available equipment, and many patients prefer SWL due to its low cost. In our center, even if three SWL sessions cost about 2400 RMB, it is still significantly cheaper than URS, which need about 7500 RMB. The SFR of re-treating ureteral calculi with SWL significantly decreases after the initial treatment and increases the complication rates of URS after unsuccessful SWL. This remind us the question whether acceptable number of SWL sessions can be followed by safe URS, regarding the intra-operative complications of URS. Studies in this area are lacking. We analyzed the influence of SWL on ureteral complications through logistic regression analysis, and showed that the more SWL sessions before URS followed by the higher risk of intra-operative complications. We calculated the optimal number of SWL sessions followed by safe URS as 0.5 with a sensitivity of 67.7% and specificity of 71.5%. We further showed that the complication rates of URS were higher in patients who suffered more than a single SWL (72.6% vs 57.4%, p = 0.047). These results suggest that no acceptable number of SWL sessions for safe URS to treat large stones in the proximal ureter currently exist. And patients who underwent more previous SWL were more likely to suffer intra-operative complications during URS complementary treatment. To get lower complications, it was suggested that URS procedures should be delayed 2–3 weeks after unsuccessful SWL [24].

This study had some limitations, including its retrospective nature that led to selection shifts. Moreover, different surgeons, the small number of patients, unpublished and missing data may have resulted in bias. However, this study was the first to identify whether acceptable number of SWL sessions exist that can be followed by safe URS for proximal ureter stones > 10 mm.

## Conclusions

The average operating time, hospitalization time, and needing further URS in complementary treatment for patients suffering SWL failure were significantly higher than primary URS lithotripsy treatment in proximal ureteral stones larger than 10 mm. There was no acceptable number of SWL sessions that could be followed by URS with fewer intra-operative complications.

## Data Availability

All data generated or analyzed during this study are included in this published article. If someone wants to request the data, please feel free to contact Feng Yao with E-mail: yaaaa.771130@163.com.

## Abbreviations

URS: Ureteroscopy
SWL: Extracorporeal shock wave lithotripsy
EAU: European Association of Urology
KUB: Kidney-ureter-bladder
CT: Computerized tomography
IVU: Intravenous urography
SFR: Stone-frees rate
